# Poisoning by Arseniuretted Hydrogen, or Hydrogen Arsenide

**Published:** 1910-03

**Authors:** 


**Poisoning by Arseniuretted Hydrogen, or Hydrogen Arsenide.** By JOHN GLAISTER, M.D. Pp. ix, 279. Edinburgh: E. & S. Livingstone. 1908.

This is a systematic investigation of permanent value, since the commercial use of metals and acids contaminated with arsenic is enormous, and the danger from the evolution of this gas in many processes is a frequent one. As the use of balloons and airships increases, accidents will become more common from the arsenic in the hydrogen gas used. It is lamentable to find how many lecturers in chemistry have been killed by the gas, besides workers in various commercial processes, such as anilin and galvanising manufactures, and in brazing by a hydrogen flame.

Besides symptoms of arsenical poisoning, this gas causes a remarkable jaundice, of a more or less coppery colour, hæmaturia, and other hemorrhages with profound alteration in the blood. The author is inclined to ascribe the jaundice to resorption, as Stadelmann does in the case of toluylendiamin poisoning, but discusses the other views which have been brought forward.

The symptoms have to be distinguished from those due to Blackwater fever, paroxysmal hæmoglobinuria, Weil's disease, and poisoning by chlorate of potash and pyrogallic acid.

The gas generated from arsenical wall-papers by the action of moulds, though not arseniuretted hydrogen, is a near ally of it—di-ethyl-arsine—and in many cases produces the remarkable jaundice of the former, and sometimes spinal troubles. One of the most valuable parts of the book is the full report of 120 cases of poisoning, with an analysis of the symptoms, collected from English and foreign authors, and a discussion of recent analytical methods.

A curious misprint occurs on page 86, where we read, "He generated sufficiently more gas to produce an explosion."

